# Supportive care needs of multicultural patients with cancer in the United Arab Emirates

**DOI:** 10.3332/ecancer.2018.838

**Published:** 2018-05-29

**Authors:** Satish Chandrasekhar Nair, Hassan Jaafar, Mohamed Jaloudi, Khaled Qawasmeh, Afra AlMarar, Halah Ibrahim

**Affiliations:** 1 Department of Academic Affairs, Tawam Hospital—Johns Hopkins Medicine Affiliate, Al Ain 15258, Abu Dhabi, United Arab Emirates; 2 Department of Medical Oncology, Tawam Hospital—Johns Hopkins Medicine Affiliate, Al Ain 15258, Abu Dhabi, United Arab Emirates; 3 Department of Nursing, Tawam Hospital—Johns Hopkins Medicine Affiliate, Al Ain 15258, Abu Dhabi, United Arab Emirates; 4 Department of Surgical Oncology, Tawam Hospital—Johns Hopkins Medicine Affiliate, Al Ain 15258, Abu Dhabi, United Arab Emirates; 5 Johns Hopkins Graduate School of Education, Baltimore, MD 21218, USA

**Keywords:** cancer, supportive care needs, Middle East, psychological needs, oncology

## Abstract

Despite the high prevalence of cancer in the Middle East, there is limited published data reporting the needs of cancer patients in this region of the world. The purpose of this study is to assess the unmet supportive care needs of oncology patients in the United Arab Emirates (UAE). From December 2014 to December 2016, a cross-sectional survey of cancer patients was conducted at a large tertiary care hospital and an oncology referral centre in the UAE, using a validated Arabic translation of the supportive care needs survey––short form (SCNS-SF34-A), assessing cancer-specific perceived needs across five domains: psychological, health system information, patient care and support, physical and daily living and sexuality. Chi-square test and Pearson’s correlation coefficient were used to assess the association between variables. Participant responses were tabulated as mean ± standard error of the mean (SEM). The response rate was 78% (210/268). Five of the 10 items from the psychological domain constituted the 10 most prevalent unmet moderate or high needs, followed by physical and daily living needs (3.04 ± 0.029, *p* < 0.001), health system information (3.03 ± 0.02, *p* < 0.001), patient care and support (2.95 ± 0.24, *p* < 0.001), with low sexuality needs (1.79 ± 0.08, *p* < 0.001). Women had significantly higher psychological unmet needs. Cultural differences were noted only in the health system information domain. Improvements in mental health services, development of multidisciplinary cancer care teams, introduction of cancer support groups and fully engaging women in all treatment decisions are feasible and easy to implement interventions that can significantly improve the care and wellbeing of oncology patients in the UAE.

## Introduction

Cancer is the second leading cause of death in the world, with over 8 million fatalities annually [1]. Despite advances in both prevention and treatment options, the cancer burden continues to rise globally, fuelled by an ageing population and increasing lifestyle-related risk factors [2]. The prevalence of cancer in the Middle East is high and growing [3]. The World Health Organization (WHO) reports that within the next 15 years, the Middle East is likely to experience the highest increase in cancer incidence among WHO regions, with predicted increases as high as 100–180% [3, 4]. Recent improvements in multi-regimen modalities have resulted in significant increases in disease-free survival rates [5, 6], but do not address the supportive care needs of oncology patients. There is a large body of literature documenting that cancer patients are at risk for adverse physical, psychological, spiritual and social problems throughout the course of their diagnosis and treatment [7]. Physical complaints, including illness and disability, psychological concerns manifested as fear of pain or death and social issues, such as family dynamics, all affect cancer patients, with levels of accompanying distress that vary from patient to patient [8]. The distress can interfere substantially with comfort, quality of life and the ability to make suitable decisions or to adhere to treatment [9]. Negative patient and family experiences can also be a reflection of health care delivery systems that do not account for patient and family needs [10]. Patient needs affect all types and stages of cancer [11]. Supportive care needs also differ across countries and cultures [12]. Among ethnically diverse groups of patients, culture has been shown to influence perceptions and coping mechanisms related to disease management [13]. Therefore, cultural beliefs and values can serve as important determinants of clinical outcomes following cancer diagnosis and treatment [14].

There is a paucity of published data and limited understanding of the unmet needs of cancer patients in the Middle East, particularly in the Gulf Cooperation Council (GCC) countries, consisting of Kuwait, Qatar, Oman, Saudi Arabia, Bahrain and the United Arab Emirates (UAE). Identifying and addressing these unmet needs can lead to many positive patient outcomes, including better ability to cope with disease symptoms and treatment side-effects, improved physician–patient communication and better adherence to treatment regimens [7, 15]. The purpose of this study is to assess the unmet needs of cancer patients in the UAE, with the goal of improving the supportive care services for these patients and, thereby, positively impacting their overall quality of life [15].

## Methods

A cross-sectional survey was conducted by a bilingual (Arabic and English) physician and, nurse researcher between December 2014 and December 2016 at a tertiary care, Joint Commission International accredited hospital and a regional oncology referral centre. The validated short form of the SCNS-SF34 was used for the study, primarily because of its feasibility and ease of use and coverage of many of the major domains of unmet needs [7]. The SCNS-SF34 assesses cancer-specific perceived needs across five analytically derived domains: psychological (10 items), health system information (11 items), patient care and support (5 items), physical and daily living (5 items) and sexuality (3 items) [16]. The SCNS-SF34 was provided by Dr. Allison Boyes (University of Newcastle upon Tyne, Australia) [16]. Prior to study commencement, the SCNS-SF34 was translated by a certified legal translator into Arabic, the preferred local language of the patients, and then, back translated into English to ensure the quality of the translation and to ensure that the structure, content and intent of the survey items did not alter during translation. Both the English (*n* = 14) and Arabic (*n* = 17) versions were pretested for reliability in the local setting using 31 patients from the oncology outpatient clinic.

Participating subjects were ambulatory patients diagnosed with cancer who presented to the oncology outpatient clinics. Potential participants were randomly selected using the patient appointment booking module of Malaffi, the hospital information system. Inclusion criteria included men or women aged 18–75 with a confirmed new or recurrent cancer diagnosis, who were informed of the cancer diagnosis and were capable of providing informed consent and completing the survey questionnaire, based on the European Cooperative Oncology Group (ECOG) status (0–3) [17]. Patient participants unable to meet the ECOG status, patients with mental or cognitive disorders and those unable to understand or unwilling to provide informed consent were excluded from the study. Respondents were asked to indicate their level of need for help during the last month for each item on the survey on a 5-point Likert scale, with the following response options: 1 = no need, not applicable; 2 = no need, satisfied; 3 = low need; 4 = moderate need and 5 = high need [18]. Survey participants who selected a score of 4 or 5 indicated moderate or high unmet needs that required assistance [16]. Additional information, if required, was obtained from the patient’s medical record and the hospital’s cancer registry. Written consents were obtained from the respondents.

Data were analysed using SPSS Statistical Software Version 20 (SPSS Inc. Chicago, USA). The domains were identified as reported elsewhere [16]. The widely used principal component analysis (PCA) was used as the extraction method to undertake exploratory factor analysis; and varimax rotation was used to rotate the factors to better fit the data [19]. Convergent validity to assess if the survey items converged to measure a construct was also conducted using the correlation coefficient matrix method [19]. The percentage of total variance by each factor was calculated and pattern matrix was used to identify the domains. Kiaser–Meyer–Olkin (KMO) sampling adequacy and Bartlett’s tests (to assess the strength of the relationship among the variables) were also applied to the construct [19]. The reliability of the inventory and its subscales were tabulated using Cronbach’s alpha. In addition to descriptive statistics, the Chi-square test was adopted to assess the association between variables. Participant responses to each of the items were tabulated as mean ± SEM. Comparison of means, when required, was accomplished using MedCalc software [20]. The study was approved by the regional research ethics committee (AAMDHREC 12/55).

## Results

Approximately 300 patients with either a new cancer diagnosis or a diagnosed recurrence are treated at these oncology outpatient clinics every year. Based on our previous experience of a fully completed survey return rate of 80% in this population [19], 34 more participants were added to the original sample size of 169 (confidence level 95%, confidence interval 5%), obtaining a minimum sample size of 203 patient participants for the study. Two hundred and ten completed questionnaires were returned out of 268 administered (78.3% response rate). The demographics of the patient participants are given in Table 1. The majority of the survey respondents were female (69%, *n* = 144), married (76%, *n* = 159), with college-level education (54%, *n* =113).

**Figure 1.**
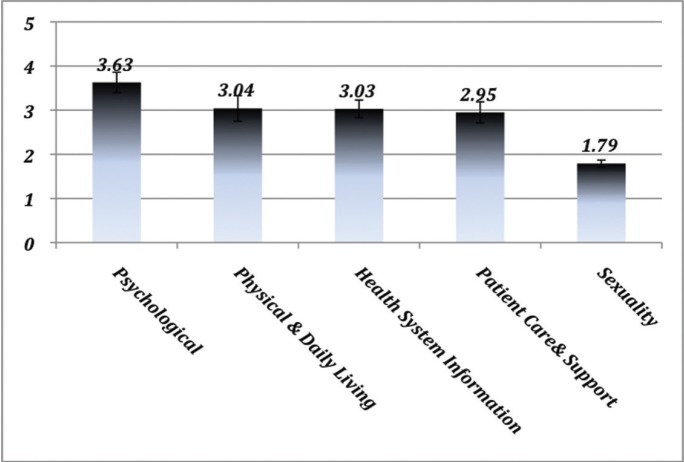
Patient responses to the SCNS-SF34-A survey are indicated as Mean ± SEM for each of the five domains: psychological, physical and daily living, health system information, patient care and support and sexuality (N = 210).

Comparison of the five analytically derived domains indicated a significantly high psychological need (3.63 ± 0.023), followed by physical and daily living needs (3.04 ± 0.029, *p* < 0.001), health system information needs (3.03 ± 0.02, *p* < 0.001), patient care and support (2.95 ± 0.24, *p* < 0.001), with low sexuality needs (1.79 ± 0.08, *p* < 0.001) (Figure 1). Five out of 10 items (50%) from the psychological domain constituted the 10 most prevalent unmet moderate or high needs of oncology patients in the UAE, including concerns regarding an uncertain future (80%) and feelings about death and dying (77.1%) (Table 2). Moderate or high feelings of lack of energy and tiredness (75.2%), as well as pain affecting their physical and daily living activities (74.8%), were prevalent in more than three-quarters of the participants. Needs related to sexuality, such as receiving information about sexual relationships (16.7%) and changes in sexual feelings (9.5%), scored low (no need or satisfied) for almost 85% of the patient population surveyed, data not shown.

Table 3 reports gender differences in participant responses. Gender correlated strongly with sexuality (changes in sexual feelings), with men expressing higher unmet need than women (*r* = 0.901, *p* < 0.001). Women had significantly higher psychological unmet needs of ‘feeling down or depressed’ (*r* = 0.42, *p* < 0.001) and ‘feelings of sadness’ (*r* = 0.26, *p* < 0.001), as compared with male respondents. The need to be informed about cancer directly from the doctor, rather than a relative, was also moderately high among women (*r* = 0.37, *p* < 0.001). Physical limitation, such as ‘feeling unwell a lot of time’, positively correlated (*r* = 0.28, *p* < 0.001) with the unmet needs for women. ‘Uncertainity about the future’ (psychological) was directly correlated with patient age (*r* = 0.14, *p* < 0.05 2-tailed significance, data not shown).

Cultural differences in responses amongst the patient respondents are givenn in Table 4. UAE nationals reported higher unmet needs in the health system information domain. No other statistically significant differences in reports of unmet supportive care needs were noted between UAE nationals and non-nationals, or between Arabs and non-Arabs (data not shown).

The consistency of the SCNS-SF-A was measured using the PCA, obtaining a KMO value of 0.88, reaching statistical significance. Bartlett’s test of sphericity was significant (0.000). The PCA revealed the presence of a component with an eigen value of 14.9, explaining 81.2% of total variance. Correlation coefficient indicating relationship between the items is given in Table 5. Cronbach’s α reliability assessment of each of the four domains ranged between 0.73 and 0.84 (Table 5). The overall Cronbach’s α reliability score for all 34 items for the final construct was high at 0.79.

## Discussion

Wellbeing in cancer patients can be considered a balance between two sets of factors: the stress and burden resulting from the cancer experience and the resources available for coping and mitigating stress and burden [10, 11]. Our study demonstrates that cancer patients in the UAE experience a wide range of unmet supportive needs, primarily related to psychological needs. Amongst the critical top 10 unmet needs of the patients surveyed, 50% were psychological in nature, with women expressing significantly higher psychological needs than men. This finding suggests that the improvement of mental health services for cancer patients is an urgent priority. Proactive encouragement and recruitment of female cancer patients into counselling services can help mitigate some of their needs.

In addition, the high prevalence of physical symptoms, including lack of energy, tiredness and pain limiting physical and daily living, is quite concerning. This highlights the need to focus on early and adequate pain management and physical rehabilitation as important components of cancer care. Oncology units in the UAE should consider inculcating these specialities into multidisciplinary cancer care teams. It has also been reported that patients have several misconceptions regarding pain, specifically that increasing pain signifies disease progression, medicine to control pain may weaken the immune system and pain is inevitable for cancer patients [21]. These beliefs identify a need for cancer support groups to address misinformation and help manage patient needs. A large body of literature demonstrates that participation in cancer support groups improves patients’ quality of life and wellbeing [22, 23]. As such, the development of this service at oncology centers throughout the UAE can serve as viable channels of information and help to meet several of the psychological needs of cancer patients.

Furthermore, the unmet need of cancer patients to be informed about their diagnostic tests in a timely manner and to be provided an explanation about their test results indicate that system changes are required. In the UAE, it is common practice to send diagnostic specimens overseas for genetic and mutational analysis, leading to delays in reporting results. The inclusion of genetic counsellors as part of the multidisciplinary care teams for cancer management can provide accurate, timely information and help alleviate some patient fears during the diagnostic process. The finding that women were more concerned about receiving test results directly from the doctor, instead of a family member, is not surprising given that the UAE, as much of the Arab world, is a patriarchal society where physicians often receive consent from, and discuss results with, male family members instead of female patients. It is important that cancer care health professionals understand the need for all patients to be included in treatment discussions. Actively involving women in all treatment decisions will help to alleviate this concern. Also, the addition of social workers into cancer care teams to directly address female patient needs may help resolve potential family dynamic issues.

Interestingly, sexuality scored low as an unmet need for cancer patients in our study, although men expressed higher unmet need for sexuality as compared to women (*r* =0.901, *p* < 0.001). This is consistent with previous studies demonstrating that patterns of unmet supportive care needs differ across cultures and health care services among Caucasians, Japanese and Chinese [24, 25, 26]. Previous studies, based on Hong Kong Chinese women with breast cancer, also showed similar low unmet needs related to sexuality [12, 13, 18, 21, 26]. In the UAE, it is not standard practice to routinely assess sexual wellbeing in cancer and palliative care patients, as it is often not a presenting symptom. Also, it is possible that the reported low sexual needs were due to an unwillingness of the patient to discuss sexual concerns. Routinely addressing sexual issues as part of cancer treatment may overcome some of these cultural barriers and ensure that this aspect of patient care needs is met.

The study did not reveal significant cultural differences in unmet supportive care needs among the oncology patients in the UAE, perhaps because most respondents were from in and around the Arab world. As all patients were residents of the UAE, it is also possible that respondents had already assimilated to UAE culture.

The strengths of this study include the high response rate and participation of multi-ethnic patients. We also attempted to minimise the effect of social desirability response bias by assuring respondent confidentiality. Our results, however, should be viewed in light of some limitations. First, the cross-sectional study design provides correlation, but not causal inference. Patients from only two institutions were surveyed; however, both participating hospitals have large oncology units and one is the regional referral centre for the UAE and neighbouring countries. Also, most patient participants had breast or gynaecologic malignancies. Perspectives of patients with other malignancies, including gastrointestinal cancers, are important. Finally, inherent to any self-report of complex issues, such as quality of life, there are influencing factors that may not have been fully addressed in this study, including family/caregiver and social support, spirituality and patient individual personality. Notwithstanding these limitations, to our knowledge, this is the first study in the region that has assessed the supportive care needs of cancer patients and provides valuable information to improve patient care. Identifying and understanding cancer patient perspectives is a first step in the development of hospital and community services to meet their needs. Future studies focusing on the implementation of integrated multidimensional approaches to cancer support services are needed to help improve the quality of UAE cancer patients’ and families’ lives.

## Conclusion

As the cancer rates climb in the UAE, it is important to fully understand and meet the needs of oncology patients. Our study has identified several opportunities to improve the care and support services available. Oncology centres in the UAE should develop an integrated, multifaceted approach to identify and meet the supportive care needs of cancer patients. The improvement of mental health services for oncology patients, development of multidisciplinary cancer care teams, introduction of cancer support groups and fully engaging women in all treatment discussions and decisions, are all feasible and easy to implement interventions that can significantly improve patient care and wellbeing. As the UAE is a multi-ethnic and multicultural society, the findings of this study may prove to be useful in addressing the needs of cancer patients in the pan Arab region.

## Conflicts of interest

The authors declare no conflicts of interest.

## Funding declaration

The study was not funded by any funding sources.

## Figures and Tables

**Table 1.** Demographics of the patient participants (N = 210).

Categories	*n*	%		Categories	*n*	%
**Gender**				**Education**		
Female	144	69		None	37	18
Male	66	31		> HighSchool	113	54
**Age**				**Children (#)**		
18–20 Y	2	1		None	27	13
21–30 Y	9	4		Less than 5	141	67
31–50 Y	99	47		5+	42	20
51–70 Y	79	38	*N* = 210	**Malignancy**		
71 + Y	18	9		Breast	126	60
**Nationality**				Gynaecological	18	9
UAE/GCC	45	21		Testicular	17	8
Middle East	95	45		Prostate	19	9
Asia	56	27		Lung	21	10
Africa	9	4		Others	9	4
**Marital status**				**Cancer stage**		
Single	28	13		I	55	26
Married	159	76		II	94	45
Others	23	11		III/IV	61	29

**Table 2.** Top ten unmet supportive care needs of cancer patients.

Moderate to high				
Domain	What was your level of need for help with:	Mean response ± SEM	*n*	%
Psychological	Uncertain about the future	4.14 ± 0.088	168	80.0
Health system information	Being informed about your test results as soon as feasible	4.0 ± 0.052	166	79.0
Psychological	Anxiety	4.28 ± 0.071	165	78.6
Psychological	Feelings about death and dying	4.14 ± 0.090	162	77.1
Physical and daily living	Lack of energy/tiredness	3.98 ± 0.082	158	75.2
Physical and daily living	Pain	4.17 ± 0.063	157	74.8
Psychological	Feelings of sadness	4.08 ± 0.079	157	74.8
Health system information	Receiving information about cancer test results to you rather than to your relative or family	4.08 ± 0.072	151	72
Health system information	Being given explanations of those tests for which you would like an explanation	3.49 ± 0.060	135	64.3
Psychological	Keeping a positive outlook	3.72 ± 0.092	129	61.4

**Table 3.** Gender differences among cancer patients reporting unmet supportive care needs.

Gender/domain	What was your level of need for help with:	Pearson correlation coeffecient{r}	Significance sig. (2-tailed)
Male/sexuality	Changes in sexual feeling	0.901	*p* < 0.001
Female/psychological	Feeling down or depressed	0.417	*p* < 0.001
Female/health system information	Receiving information about cancer test results to you rather than to your relative or family	0.378	*p* < 0.001
Female/physical and daily living	Feeling unwell a lot of time	0.281	*p* < 0.001
Female/psychological	Feelings of sadness	0.261	*p* < 0.001

**Table 4.** Comparison between UAE nationals and non-nationals (expatriates), cancer patients reporting unmet supportive care needs.

Domain	Items	Population	Moderate/high need *n*(%)	*P* Value
Health system information	Being given explanations of those tests for which you would like an explanation	UAE nationals (*n* = 47)	36 (76)	0.023
		Expatriates (*n* = 158)	95 (61)	
Health system information	Being treated like a person not just another case	UAE nationals (*n* = 47)	14 (30)	0.001
		Expatriates (*n* = 158)	25 (16)	

**Table 5.** Reliability and validity of the SCNS34-A construct. Internal consistency of the inventory and its subscales were tabulated using Cronbach’s alpha and PCA was used as the extraction method to undertake factor analysis.

#	Domain	Items	Correlation coeffcient	Cronbach’s alpha
1	Physical and daily living	Pain	0.633	0.78
2	Physical and daily living	Lack of energy/tiredness	0.791	
3	Physical and daily living	Feeling unwell a lot of time	0.641	
4	Physical and daily living	Work around the home	0.711	
5	Physical and daily living	Not being able to do things that you used to do	0.643	
6	Psychological	Anxiety	0.661	0.81
7	Psychological	Feeling down or depressed	0.546	
8	Psychological	Feelings of sadness	0.627	
9	Psychological	Fears about cancer spreading	0.702	
10	Psychological	Worry that results of treatment are beyond your control	0.636	
11	Psychological	Uncertain about the future	0.701	
12	Psychological	Learning to feel in control of your situation	.536	
13	Psychological	Keeping a positive outlook	0.684	
14	Psychological	Feelings about death and dying	0.651	0.73
15	Psychological	Concerns about the worries of those close to you	0.528	
16	Sexuality	Changes in sexual feeling	0.907	
17	Sexuality	Changes in your sexual relationships	0.914	
18	Sexuality	Being given information about sexual relationships	0.606	
19	Patient care and support	More choice about which cancer specialists you see	0.637	0.84
20	Patient care and support	More choice about available best treatment for your disease	0.658	
21	Patient care and support	Reassurance by medical staff that the way you feel is normal	0.629	
22	Patient care and support	Hospital staff attending promptly to your physical Needs	0.767	
23	Patient care and support	Hospital staff acknowledging, showing sensitivity to your feelings and emotional needs	0.717	
24	Health system information	Being given written information about the important aspects of your care	0.752	
25	Health system information	Being given information (written, diagrams and drawings) about aspects of managing your illness and side effects at home	0.638	
26	Health system information	Being given explanations of those tests for which you would like an explanation	0.568	
27	Health system information	Being adequately informed about the benefits and side effects of treatments before you choose to have them	0.521	
28	Health system information	Being informed about your test results as soon as feasible	0.493	
29	Health system information	Being informed about cancer to you rather than to your relative or family	0.433	
30	Health system information	Being informed about things you can do to help yourself to get well	.448	
31	Health system information	Having access to professional counselling (examples: psychologist, social worker, counsellor and nurse specialist) if you, family or friends need it	0.504	0.8
32	Health system information	Being treated like a person not just another case	0.635	
33	Health system information	Being treated in a hospital or clinic that is as physically pleasant as possible	0.736	
34	Health system information	Having one member of hospital staff with whom you can talk to about all aspects of your condition, treatment and follow-up	0.639	
